# Structural insights and *ab initio* sequencing within the DING proteins family

**DOI:** 10.1107/S0909049510036009

**Published:** 2010-11-05

**Authors:** Mikael Elias, Dorothee Liebschner, Guillaume Gotthard, Eric Chabriere

**Affiliations:** a Weizmann Institute of Science, Rehovot, Israel; b CRM2, Nancy Université, France; c AFMB, Université Aix-Marseille II, France

**Keywords:** serendipity, DING protein, *ab initio* sequencing, sub-angstrom crystallography, HIV inhibition

## Abstract

DING proteins constitute an intriguing family of phosphate-binding proteins that was identified in a wide range of organisms, from prokaryotes and archae to eukaryotes. Despite their seemingly ubiquitous occurrence in eukaryotes, their encoding genes are missing from sequenced genomes. Such a lack has considerably hampered functional studies. In humans, these proteins have been related to several diseases, like atherosclerosis, kidney stones, inflammation processes and HIV inhibition. The human phosphate binding protein is a human representative of the DING family that was serendipitously discovered from human plasma. An original approach was developed to determine *ab initio* the complete and exact sequence of this 38 kDa protein by utilizing mass spectrometry and X-ray data in tandem. Taking advantage of this first complete eukaryotic DING sequence, a immunohistochemistry study was undertaken to check the presence of DING proteins in various mice tissues, revealing that these proteins are widely expressed. Finally, the structure of a bacterial representative from *Pseudomonas fluorescens* was solved at sub-angstrom resolution, allowing the molecular mechanism of the phosphate binding in these high-affinity proteins to be elucidated.

## Abstract 2

DING proteins constitute a recently discovered protein family that is ubiquitous in eukaryotes. The structural insights and the physiological involvements of these intriguing proteins are hereby deciphered.

## 1. The DING proteins

DING proteins constitute an intriguing family of phosphate-binding proteins named DING according to their four conserved N-terminal residues (Berna *et al.*, 2002 ▶). Surprisingly, the genes coding for these proteins are systematically missing from eukaryotic sequenced genomes, despite the fact that these proteins seem ubiquitous in eukaryotes, being isolated in animals (human, monkey, rat, turkey), in plants (*Arabidopsis thaliana*, potato, tobacco) and in fungi (*Candida albicans*, *Ganoderma lucidum*) (Berna *et al.*, 2002 ▶; Riah *et al.*, 2000 ▶; Belenky *et al.*, 2003 ▶; Blass *et al.*, 1999 ▶; Kumar *et al.*, 2004 ▶; Adams *et al.*, 2002 ▶; Weebadda *et al.*, 2001 ▶; Scott & Wu, 2005 ▶; Morales *et al.*, 2006 ▶; Du *et al.*, 2007 ▶; Chen *et al.*, 2007 ▶). Furthermore, the DING proteins family extends to prokaryotes (Berna *et al.*, 2008 ▶), as some representatives and their corresponding genes have been identified in *Pseudomonads* (Ahn *et al.*, 2007 ▶), whereas in some other bacteria the encoding gene remains unidentified (Pantazaki *et al.*, 2008 ▶). In eukaryotes, partial DNA sequences coding for this protein family have been cloned or identified in unannotated parts of genomes (Berna *et al.*, 2008 ▶; Berna, Bernier *et al.*, 2009 ▶), and another interesting point in genetics concerns the sequence conservation. Indeed, between distant species such as potato (a higher plant) and *Leishmania major* (a protozoan) the sequence identity between the known DING representatives is about 90% at the nucleotidic level, over more than 600 base pairs (Morales *et al.*, 2006 ▶). This high conservation raised controversy about their prokaryotic (Lewis & Crowther, 2005 ▶) or eukaryotic origins (Berna, Scott *et al.*, 2009 ▶).

DING proteins have been mostly isolated by virtue of a biological function. One of the best illustrations is the search for a new HIV inhibitor in St John’s wort that led to the characterization of a novel DING protein named p27^sj^ (Darbinian-Sarkissian *et al.*, 2006 ▶). In humans, several DING proteins have been identified from different tissues, including the crystal adhesion inhibitor (CAI), the human synovial stimulatory protein (SSP), X-DING-CD4+ from human CD4+ T lymphocytes and the human phosphate binding protein (HPBP). Comparison of available peptides sequences of HPBP, CAI, SSP and X-DING-CD4+ strongly suggests that these proteins are encoded by four different genes, all lacking the sequenced human genome. The CAI, isolated from human kidney cells, is assumed to prevent the growth of kidney stones (Kumar *et al.*, 2004 ▶). The SSP, isolated from human synovial liquid, possesses auto-antigen activity, lymphocyte stimulatory activity and a putative role in the etiology of rheumatoid arthritis (Hain *et al.*, 1990 ▶, 1996 ▶). X-DING-CD4+ was isolated from CD4+ T cells that are resistant to HIV infection and was shown to block the HIV-1 LTR promoted expression and the replication of HIV-1 (Lesner *et al.*, 2009 ▶). HPBP is a serendipitously discovered plasma lipoprotein that binds phosphate and was isolated from human plasma (Fokine *et al.*, 2003 ▶; Contreras-Martel *et al.*, 2006 ▶). HPBP structure was solved (Morales *et al.*, 2006 ▶) and its physiological function, *i.e.* its association with the paraoxonase (HPON1), an enzyme involved in atherosclerosis (Shih *et al.*, 1998 ▶), has been extensively studied (Renault *et al.*, 2010 ▶; Rochu *et al.*, 2008 ▶; Rochu, Renault *et al.*, 2007 ▶; Rochu, Chabriere *et al.*, 2007 ▶). The involvement of DING proteins in a large spectrum of diseases enhances the potential therapeutic value of this specific protein family, but the lack of sequences has considerably hampered the functional studies within this protein family.

## 2. *Ab initio* sequencing of HPBP

HPBP is a plasmatic protein interacting with HPON1 and possibly involved in inflammation and atherosclerosis processes (Webb, 2006 ▶). HPBP was serendipitously discovered while performing structural studies on supposedly pure HPON1 samples purified from human plasma. Crystals were obtained and the resolved structure was not that of HPON1 but rather that of an unexpected and unknown protein: HPBP. As for other DING proteins, the lack of genetic sequence encoding for HPBP has considerably hindered functional studies. In order to overcome this difficulty, HPBP’s sequence was determined experimentally. However, the *ab initio* sequencing of a protein of 38 kDa is not a trivial task, and can barely be achieved using only one technique, *i.e.* mass spectrometry, mainly because some of the protein peptides are too hydrophobic and barely observed in this experiment. A new strategy was developed, utilizing mass spectrometry sequencing and available X-ray data in tandem (Diemer *et al.*, 2008 ▶).

### 2.1. Limitations of the X-ray sequencing

**Figure 1:**
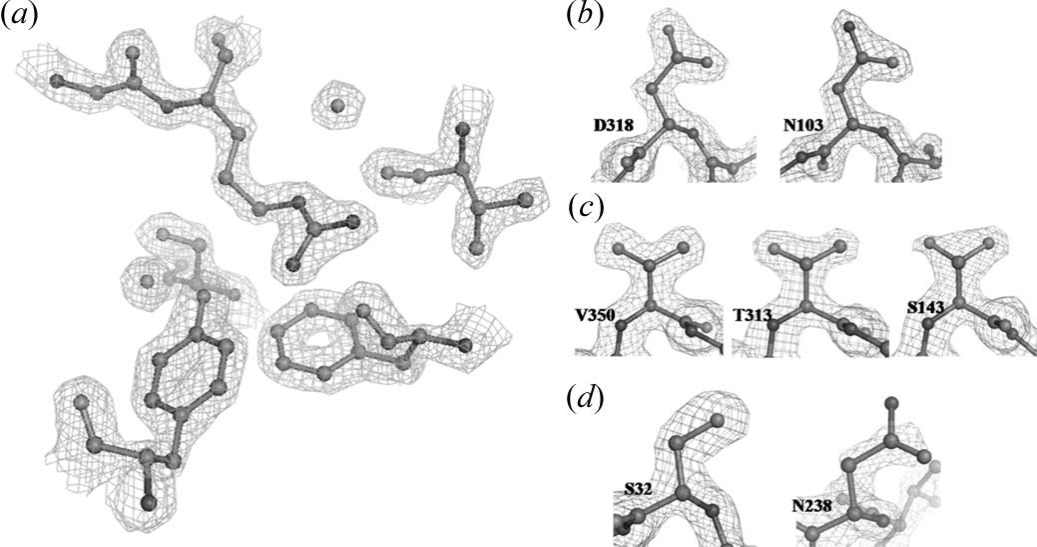
(*a*) Close view of a ball-and-stick representation of the R374 region in the HPBP structure at 1.9 Å resolution. The 2*f*_obs_ − *f*_calc_ electronic density map is contoured at 1.75σ. (*b*) Comparison between the electronic density shapes of N103 and D318. The 2*f*_obs_ − *f*_calc_ electronic density map is contoured at 1.5σ. (*c*) Comparison between electronic density shapes of V350, T313 and S143. The 2*f*_obs_ − *f*_calc_ electronic density map is contoured at 1.5σ. (*d*) Comparison between the electronic density shapes of N238 and S32. The 2*f*_obs_ − *f*_calc_ electronic density map is contoured at 1.5σ.

The first HPBP sequence was inferred from electronic density maps at 1.9 Å (Fig. 1*a* ▶). However, this sequence contains some ambiguities. The electronic density map is related to the electron number of the atoms, but at this resolution it is not possible to clearly discriminate C, N and O atoms as they possess roughly the same number of electrons (six, seven and eight electrons, respectively). This limitation implies that some amino acids possess similar electronic density shapes at such a resolution, such as Asn and Asp, Gln and Glu, and Val and Thr (Fig. 1*b* ▶), and are thus difficult to discriminate. Furthermore, some protein residues possess multiple conformations. Agitation modifies the electronic density shape. As an illustration, a double serine conformation causes similar electronic density shapes as threonine or valine residues (Fig. 1*c* ▶). A third cause of ambiguity concerns dis­ordered atoms. Indeed, disordered atoms contribute less than ordered atoms in diffraction. Consequently, these agitated atoms disappear from the electronic density maps. This mainly concerns residues located at the protein extremities or surface, and causes truncated electronic density, which can be assimilated to the density corresponding to shorter residue (Fig. 1*d* ▶).

### 2.2. Combination of X-ray data and mass spectrometry data

A series of enzymatic digestions was performed on HPBP to generate peptides, allowing a maximum of sequence information by mass spectrometry (MS) fragmentation in LC-MS/MS and MALDI-MS/MS experiments to be obtained. The primary sequence obtained by X-ray crystallography was used like an ‘Ariane wire’, useful to align peptide sequences subsequently obtained by mass spectrometry, without the need of having overlapping peptides. It can be noted that X-ray crystallography techniques provided important information that can barely be obtained using MS, such as the exact number of amino acids and the presence of the disulfide bridges, and the discrimination of residues that possess the same mass (Leu, Ile, *etc.*). MS experiments, including ESI-MS on intact HPBP, were used to correct errors from crystallographic sequencing, including those for the few peptides that could not be sequenced. Finally, this technique allowed, *ab initio* and without ambiguities, the 38 kDa HPBP to be sequenced (Diemer *et al.*, 2008 ▶), showing that this method could be applied to other DING proteins.

## 3. The tissue localization of DING proteins

Taking advantage of obtaining the HPBP sequence (Diemer *et al.*, 2008 ▶), several polyclonal and monoclonal antibodies targeted against HPBP were developed. Because of the very high sequence identity between DING proteins sequences, the polyclonal antibodies are able to cross-react with other DING proteins. This property was used to map the DING proteins localization in several mouse tissues by immunohistochemistry.

DING proteins were observed in all tested tissues, namely brain, skin, heart, aorta, lung and liver, suggesting that these proteins are widely expressed within the organism (Collombet *et al.*, 2010 ▶). A western blot study on these samples also confirms previous assumptions, stemming from the partial gene found in *Leishmania* major genome and western blot studies on plant tissues (Perera *et al.*, 2008 ▶), suggesting that DING proteins exists also as high-molecular-weight proteins (HMW-DING). Indeed, if most of the characterized DING proteins are 38 kDa proteins, our western blot study shows that several HMW-DINGs exists, such as the 140 kDa, the 71 kDa, the 62 kDa and the 52 kDa DING (Collombet *et al.*, 2010 ▶). The presence of several isoforms of DING proteins might be linked with different biological activities. Indeed, it was shown for a bacterial DING representative named PfluDING that the truncated form possesses higher stimulation effects on human fibroblasts proliferation than the 38 kDa form (Ahn *et al.*, 2007 ▶). This result suggests that there is still a lot to do to understand the physiological involvements of these putatively uncharacterized proteins.

The immunohistochemistry study also reveals that the DING protein cellular localization is tissue-dependent, being exclusively nuclear in neurons, and nuclear and cytoplasmic in the heart muscle. The nuclear localization of DING proteins fits well with previous observations concerning biological activities of DING proteins, showing a clear involvement of these proteins in complex processes within the nucleus. For example, p27^SJ^ suppresses expression of HIV-1 genome (Darbinian *et al.*, 2008 ▶). This suppression of expression is mediated by the physical and functional association of p27^SJ^ with human C/EBPβ transcription factor and viral Tat transactivator. Moreover, p27^SJ^ possesses a phosphatase activity inducing a dysregulation at S and G2/M phases in cell cycles related to alteration of the Erk1/2 phosphorylation state (Darbinian *et al.*, 2009 ▶). In addition, X-DING-CD4+ seems to interact with transcription factors in the nucleus, and is believed to be involved in the resistance to HIV infection of non-progressive patients (Lesner *et al.*, 2005 ▶, 2009 ▶).

## 4. The structure of DING proteins

**Figure 2:**
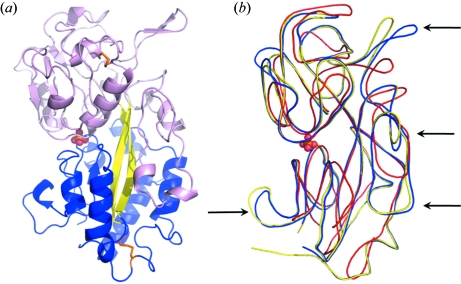
(*a*) X-ray structure of HPBP. The two globular domains are shown in pink and blue. They are hinged by an antiparallel two-stranded β-sheet acting as a hinge (in yellow), and form a deep cleft wherein a phosphate molecule is bound (red balls). The two disulfide bridges (C113–C158 and C306–C359) are shown by orange sticks. (*b*) Structural comparison of different known phosphate-SBPs: HPBP [Protein Data Bank (PDB) ID: 2v3q] is shown in blue, PfluDING (PDB ID: 2q9t) is shown in yellow, *E. coli* PstS protein (PDB ID: 1ixh) is shown in red. The four protruding DING protein-specific loops are indicated by black arrows.

Two structures of DING representatives are available: the structure of HPBP (Morales *et al.*, 2006 ▶) and the structure of a bacterial representative from *Pseudomonas fluorescens* called PfluDING (Ahn *et al.*, 2007 ▶; Moniot *et al.*, 2007 ▶). These two structures confirm the ability of these proteins to bind a single phosphate ion, in the same manner as the bacterial pstS, which sequesters phosphate for cellular uptake by the ABC phosphate transporter. These two structures and the pstS fit a model known as the ‘Venus flytrap’, in which the structure can adopt an open and a closed form depending on the phosphate binding (Luecke & Quiocho, 1990 ▶). The DING proteins structures reveal an elongated fold composed of two globular domains (Fig. 2*a* ▶). Each domain constitutes a central β-sheet core flanked by α-helices and contains a disulfide bridge that is conserved among the family. Interconnected by an antiparallel two-stranded β-sheet acting as a hinge, the two domains form a deep cleft wherein a phosphate molecule is bound. This fold, known as the Venus flytrap, is very similar to those of the sixth family of solute binding proteins (SBP) (Felder *et al.*, 1999 ▶). Structural superposition shows a high correspondence between PfluDING, HPBP and the *Escherichia coli* phosphate-binding protein. Interestingly, the unique feature of DING proteins compared with pstS is the presence of four protruding loops at the protein surface (Fig. 2*b* ▶).

## 5. Elucidation of the phosphate-binding mechanism

Although their phosphate-binding ability has not been clearly related to their biological functions until now, DING proteins are able to bind phosphate with high affinity. Indeed, it has been shown that HPBP and PfluDING bind phosphate with a *K*_D_ of approximately 1 µ*M* (Ahn *et al.*, 2007 ▶; Luecke & Quiocho, 1990 ▶), of the same order as bacterial phosphate solute binding protein (Poole & Hancock, 1984 ▶; Luecke & Quiocho, 1990 ▶). As PfluDING yields crystals diffracting to very high resolution, it offers the most convenient model for investigating the molecular mechanism of the phosphate binding in these high-affinity binding proteins. The sub-angstrom resolution structures of PfluDING (0.98 Å and 0.88 Å) at two different pH values (4.5 and 8.5) have been successfully obtained (Liebschner *et al.*, 2009 ▶).

**Figure 3:**
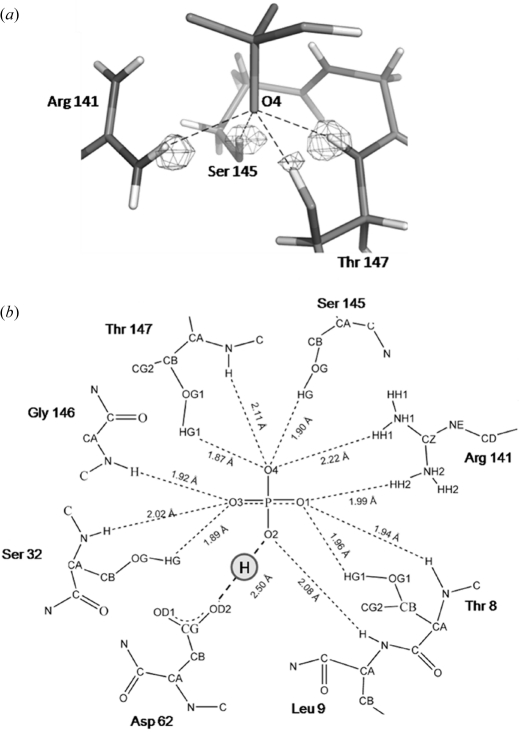
(*a*) Close view of phosphate O4 in the PfluDING structure obtained at pH 4.5. The *F*_obs_ − *F*_calc_ map is contoured at 2.6σ. (*b*) Experimentally determined hydrogen bond network involving the phosphate molecule bound in the PfluDING structure.

The quality of the obtained data allows most of the H atoms in the protein structure to be located precisely (Fig. 3*a* ▶). Moreover, the H atoms involved in the binding of the phosphate ion are clearly visible in both structures. Surprisingly, and despite the intrinsic p*K*_a_ values of the phosphate moiety, PfluDING binds only dibasic phosphate both at acidic and basic pH. The structures show that the phosphate ion is bound *via* 11 normal hydrogen bonds plus a highly energetic hydrogen bond, between a phosphate oxygen and the carboxylate side chain of Asp62 (Fig. 3*b* ▶). This very short bond (2.50 Å) belongs to the low barrier hydrogen bond (LBHB) type, where the H atom is almost perfectly shared between the two heavy atoms. This work, combined with electrostatic potential computations, demonstrates the capacity of the protein to alter the p*K*_a_ of atoms in the binding site. Indeed, the fact that PfluDING binds only dibasic phosphate both at acidic and basic pH can be explained by the finding of a very positively charged binding site, capable of altering dramatically the phosphate p*K*_a_.

## 6. Conclusion

The DING proteins family is an intriguing protein family that seems ubiquitous in eukaryotes, albeit their coding genes are missing. This unconventional protein family requires, for its investigation, some methodological developments. For example, an original approach was developed in order to sequence *ab initio* HPBP using mass spectrometry and X-ray data in tandem. Taking advantage of the very high diffracting power of DING protein crystals, we elucidated the molecular mechanism of phosphate binding in high-affinity proteins. These studies illustrate that DING proteins are widely expressed in eukaryotic tissues, and their cellular localization is tissue-dependent, albeit being mostly nuclear. This nuclear localization partly explains some observed biological activities, such as the role in the cell cycle and the inhibition of the HIV replication by interacting with the viral protein Tat and the human transcription factor CEBP/β. The involvement of DING proteins in several important human diseases, together with their genetic mystery and our findings of unknown HMW-DING in eukaryotes, enhance the emerging scientific interest on this protein family.
